# To the Skin and Beyond: The Immune Response to African Trypanosomes as They Enter and Exit the Vertebrate Host

**DOI:** 10.3389/fimmu.2020.01250

**Published:** 2020-06-12

**Authors:** Omar A. Alfituri, Juan F. Quintana, Annette MacLeod, Paul Garside, Robert A. Benson, James M. Brewer, Neil A. Mabbott, Liam J. Morrison, Paul Capewell

**Affiliations:** ^1^Roslin Institute, Royal (Dick) School of Veterinary Studies, University of Edinburgh, Edinburgh, United Kingdom; ^2^Wellcome Centre for Integrative Parasitology, College of Medical, Veterinary and Life Sciences, Institute of Biodiversity, Animal Health and Comparative Medicine, University of Glasgow, Glasgow, United Kingdom; ^3^College of Medical, Veterinary and Life Sciences, Institute of Biodiversity, Animal Health and Comparative Medicine, University of Glasgow, Glasgow, United Kingdom

**Keywords:** African trypanosomiasis, *Trypanosoma brucei*, skin, transmission, innate immunity, neglected tropical disease

## Abstract

African trypanosomes are single-celled extracellular protozoan parasites transmitted by tsetse fly vectors across sub-Saharan Africa, causing serious disease in both humans and animals. Mammalian infections begin when the tsetse fly penetrates the skin in order to take a blood meal, depositing trypanosomes into the dermal layer. Similarly, onward transmission occurs when differentiated and insect pre-adapted forms are ingested by the fly during a blood meal. Between these transmission steps, trypanosomes access the systemic circulation of the vertebrate host *via* the skin-draining lymph nodes, disseminating into multiple tissues and organs, and establishing chronic, and long-lasting infections. However, most studies of the immunobiology of African trypanosomes have been conducted under experimental conditions that bypass the skin as a route for systemic dissemination (typically *via* intraperitoneal or intravenous routes). Therefore, the importance of these initial interactions between trypanosomes and the skin at the site of initial infection, and the implications for these processes in infection establishment, have largely been overlooked. Recent studies have also demonstrated active and complex interactions between the mammalian host and trypanosomes in the skin during initial infection and revealed the skin as an overlooked anatomical reservoir for transmission. This highlights the importance of this organ when investigating the biology of trypanosome infections and the associated immune responses at the initial site of infection. Here, we review the mechanisms involved in establishing African trypanosome infections and potential of the skin as a reservoir, the role of innate immune cells in the skin during initial infection, and the subsequent immune interactions as the parasites migrate from the skin. We suggest that a thorough identification of the mechanisms involved in establishing African trypanosome infections in the skin and their progression through the host is essential for the development of novel approaches to interrupt disease transmission and control these important diseases.

## Introduction

African trypanosomiasis has historically been the cause of large outbreaks of human disease, likely contributing to the deaths of millions of people across sub-Saharan Africa in the early twentieth century (1, 2) and inflicting substantial economic damage on the African agriculture industry to this day (3, 4). African trypanosomes include an array of vector-borne, single cell hemoflagellate protozoa (order *Kinetoplastida*), although three species cause major disease: *Trypanosoma brucei, T. congolense*, and *T. vivax. Two* subspecies of *T. brucei, T. b. rhodesiense*, and *T. b. gambiense*, cause human African trypanosomiasis (HAT), also known as sleeping sickness (5–7), with more than 57 million people at risk of infection (6, 8). *T. congolense, T. vivax*, and *T. brucei* are also the most significant contributors to disease in livestock animals (animal African trypanosomiasis or AAT). Classically, the trypanosome lifecycle starts with the tsetse fly (*Glossina* spp.) depositing an inoculum of metacyclic trypomastigotes in the skin when taking a blood meal (9–11). Following intradermal (i.d.) inoculation of metacyclic forms, the parasites differentiate into long-slender trypomastigotes that are proliferative and able to establish patent infections in the vertebrate host. However, the timing and the mechanisms controlling these events remain unclear (3, 12, 13). From the initial site of infection, the proliferative long-slender form trypanosomes travel to the local draining lymph nodes *via* afferent lymphatic vessels before disseminating systemically and establishing a patent infection in the bloodstream (14–17). African trypanosomes (and *T. brucei* in particular) also actively colonize multiple tissues in the vertebrate host, including the skin. Skin-dwelling parasites functionally and behaviorally adapt to their microenvironment, allowing them to thrive and persist (18, 19). These recent studies demonstrate that there is a previously underappreciated heterogeneity in the population of parasites residing within the vertebrate host, with important implications for understanding the biology of trypanosomes and the way in which the host responds to infection. The presence of trypanosomes in the skin has been demonstrated in both animal models of infection and human clinical samples, suggesting that it is a central aspect of transmission. Nonetheless, the mechanisms deployed by trypanosomes to inhabit and migrate from the cutaneous environment, and the interplay between resident skin cells (including immune cells), and trypanosomes during the onset of the infection, remain largely unexplored. In this review, we aim to (i) highlight current knowledge on trypanosome establishment of infection in the skin; (ii) examine the interactions between the host immune system and trypanosomes in the skin; (iii) explore the mechanisms of trypanosome migration from the skin toward systemic infection and further transmission; and finally (iv) discuss the potential of novel therapeutic and intervention strategies being developed as a consequence of these studies.

### Skin as the Initial Barrier—From Immune Response to Systemic Dissemination

**Figure 1:**
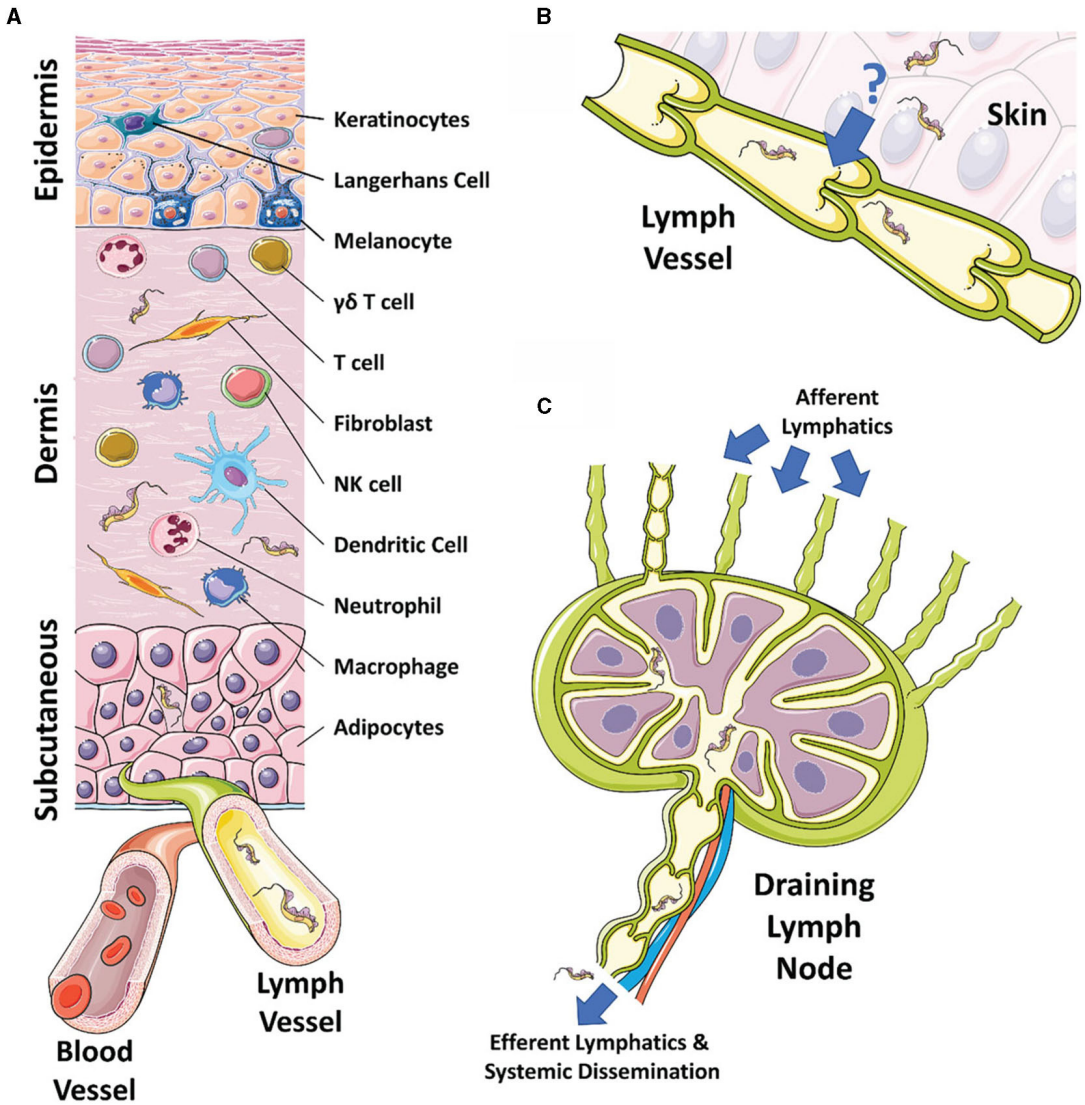
The skin, draining lymphatics, and lymph nodes. **(A)** Diagram of the cellular composition of the epidermal, dermal, and subcutaneous layers of mammalian skin. The outermost epidermal layer consists of a layer of corneocytes above a layer of keratinocytes. These cells manage the tight junctions and the stratum corneum. Langerhans cells and intraepithelial T cells survey the epidermis for antigen to be presented. The central dermal layer contains fibroblasts that produce extracellular matrix proteins to provide structural support and elasticity. Immune responses are initiated by dermal macrophages, dermal dendritic cells, NK cells, and T cells. The inner subcutaneous layer primarily consists of adipocytes. Local lymphatic and blood vessels allow for the trafficking of cells, proteins, and waste. The initial tsetse fly bite injects trypanosomes into the dermis. From the dermis, the parasites exhibit tropism that leads to migration toward the afferent lymph vessels in the skin disseminating to the blood and other regions of the body. **(B)** The mechanism behind directional migration of trypanosomes from the skin to the lymphatics is unknown. Parasites may be responding to an unreported chemical cue in a chemotactic manner and they may crawl along lymph vessels, access open junctions, or are drawn into the lymphatics through hydrodynamic flow force and pressure. **(C)** Afferent lymphatic vessels in the skin allow for the drainage of leukocytes and antigen into the draining lymph node. Lymph, containing activated T and B cells, plasma cells, and antibody, passes into the medullary sinus, before exiting via efferent lymphatic vessels. Trypanosomes enter the draining lymph nodes, causing lymphadenopathy, and exit via the efferent lymphatics. Systemic dissemination of the host is reached via the main lymphatic ducts.

Upon infection, metacyclic trypomastigotes must circumvent several environmental challenges in order to develop into the proliferative long-slender form trypomastigotes. This series of events ultimately leads to parasite dissemination in the host bloodstream but involves interactions between the developmental stages of the parasite, the host cells in the dermis, and the immune cells recruited to the site of infection. Mammalian skin is a large, highly complex organ that acts as a protective barrier between the internal components of the host and the external environment (Figure 1) (20, 21). The mechanism by which the skin protects the host is not simply through providing a physical barrier, but also the collection of immune cells, biological factors, layers of tissue, and networks of lymphatic and blood vessels (21–23). The three main components of the skin are the epidermis, dermis, and subcutaneous layer, each containing various immune cells involved in innate responses, inflammation, and surveillance (Figure 1A) (21, 24). The dermis is primarily connective tissue produced by dermal fibroblasts. Local immune responses generated within the tissue are initiated by dermal macrophages, dermal dendritic cells, natural killer (NK) cells, mast cells, αβ/γδ T cells, and natural killer T (NKT) cells (25, 26). The skin also contains numerous blood and lymphatic vessels, nerves, and (in humans but not mice) sweat glands (23, 27, 28). Together, these layers create a highly organized body compartment that represents a strong physical and biological barrier to pathogens and systemic infections.

### The Tsetse Fly Vector, the Feeding Bite, and the Site of Infection

Within an infected tsetse fly, *T. brucei* undergoes a range of complex developmental stages (29). When first taking a blood-meal from an infected mammalian host, parasites are ingested, and become proliferative procyclic trypomastigote forms in the midgut of the tsetse fly. These procyclic trypomastigotes divide rapidly before transforming into mesocyclic forms in the alimentary tract that then invade the salivary glands and transform into the rapidly proliferating epimastigotes. Tsetse saliva provides an environment that promotes trypanosome adherence to epithelial surfaces, the initiation of binary fission, and also triggers their transformation into mammal-infective metacyclic trypomastigotes (29–31). Without the presence of these salivary components, metacyclic trypomastigotes have reduced infectivity (32). Following an infected tsetse fly feeding on a mammalian host, trypanosomes are deposited into the dermis of the host skin (11). During this process, the tsetse fly proboscis inflicts significant trauma on the skin and associated tissues, while also introducing a concoction of active compounds via the saliva (33). In humans, a trypanosome-filled lesion known as a chancre often develops 5–15 days post-inoculation, which comprises indurated and inflamed patches of skin (34, 35). Similar lesions also occur in goats and cattle (36). The development of a chancre may be related to several components of tsetse saliva that have been shown to affect inflammation at the site of infection, including 5′nucleotidase-related (5′Nuc), tsetse thrombin inhibitor (TTI), and both thrombin serine protease, and esterase inhibitors (30, 37–40). This altered immune state is characterized by elevated interleukin IL-4 and IL-10 production (30, 32, 40–42). Murine models of infection have also shown that tsetse saliva suppresses T and B cell responses systemically, skewing the host toward a Th2 immune environment, leading to increased IL-4 and IL-10, alongside decreased interferon-γ (IFN-γ) titers, in the draining lymph nodes of infected mice. There is also an associated immunoglobulin (Ig) IgG1 and IgE antibody response. In addition, bites from tsetse flies infected with trypanosomes showed significantly reduced thrombin inhibition and less anticoagulation compared to bites by naïve tsetse flies. This results in a more prolonged feeding period that contributes to an increased likelihood of parasite transmission (9). Together, these anti-inflammatory and anti-coagulative processes act to increase the efficiency of parasite transmission from the fly vector to the mammalian host (32), leading to the induction of microenvironmental conditions more suitable for metacyclic trypomastigotes. This facilitates their differentiation into proliferative long-slender trypomastigotes. In this context, it appears likely that metacyclic trypomastigotes are equipped with various molecular tools (e.g., secreted virulence factors) to initiate tissue colonization events and overcome the acute response elicited by skin-resident and recruited immune cells. This leads to a scenario in which the acute immune response at the site of infection may act as a bottleneck that selects for more infectious parasites. Consequently, the route of infection could potentially shape downstream interactions and responses in other body compartments, affecting parasitaemia and longer-term infection dynamics.

### Influence of the Infection Route for Trypanosome Dissemination

Despite the skin being the first point of contact between metacyclic trypomastigotes and the vertebrate host, the importance of the skin-stage of disease in trypanosome infections has largely been overlooked in experimental studies. The majority of prior experiments have examined infections initiated *via* needle challenge, predominantly by intraperitoneal (i.p.) or intravenous (i.v.) routes (Table 1). However, a small number of comparative experiments have revealed that inoculation routes can substantially affect outcome (43, 44). For example, it has been shown that the percentage of BALB/c mice with detectable parasitemia after infection by *T. brucei* differed between animals infected i.p. vs. i.d. This study also found that the proportions of mice displaying detectable parasitemia were significantly reduced in the i.d. infected mice compared to i.p. infected mice, and that i.d. infected mice were 100-fold less susceptible to trypanosome infection than i.p. infected mice in a dose-dependent manner (43). Moreover, the impact of infection route on infectivity also differs between trypanosome species (44). For example, *T. congolense* infected intramuscularly (i.m.) had an earlier onset of parasitemia compared to those infected *via* an i.d. route. However, animals in which *T. brucei* parasites were administered i.p. led to an earlier onset of parasitemia than both i.m. and i.d. administration. These observations suggest that parasites face tissue-specific challenges in the skin (e.g., resident immune cells or nutrient availability) that delineate their capacity to disseminate systemically within the vertebrate host (44).

**Table 1:** Susceptibility of various animal models to different trypanosome strains represented by the proportion of animals displaying a patent infection, depending on route and dose.

**Trypanosome species and strain**	**Mammalian host species**	**Animal strain/breed**	**Inoculation route**	**10^**6**^ dose**	**10^**5**^ dose**	**10^**4**^ dose**	**10^**3**^ dose**	**10^**2**^ dose**	**References**
*T. b. brucei*, strain 10–26	Mouse	BALB/c	i.p.	–	100%	100%	100%	100%	(43)
*T. b. brucei*, strain 10–26	Mouse	BALB/c	i.d.	–	100%	100%	50%	0%	
*T. congolense, Trans Mara*, TC13	Mouse	BALB/c	i.p.	–	–	100%	100%	100%	
*T. congolense, Trans Mara*, TC13	Mouse	BALB/c	i.d.	–	–	100%	100%	0%	
*T. b. brucei*, strain 10–26	Mouse	C57BL/6	i.p.	–	100%	100%	100%	100%	
*T. b. brucei*, strain 10–26	Mouse	C57BL/6	i.d.	–	100%	100%	50%	0%	
*T. congolense, Trans Mara*, TC13	Mouse	C57BL/6	i.p.	–	100%	100%	100%	100%	
*T. congolense, Trans Mara*, TC13	Mouse	C57BL/6	i.d.	–	–	100%	100%	0%	
*T. b. brucei*, KETRI 2710	Mouse	Swiss white	i.p.	–	–	100%	–	–	(44)
*T. b. brucei*, KETRI 2710	Mouse	Swiss white	i.v.	–	–	100%	–	–	
*T. b. brucei*, KETRI 2710	Mouse	Swiss white	i.m.	–	–	100%	–	–	
*T. b. brucei*, KETRI 2710	Mouse	Swiss white	s.c.	–	–	100%	–	–	
*T. b. brucei*, KETRI 2710	Mouse	Swiss white	i.d.	–	–	83%	–	–	
*T. congolense*, KETRI 2765	Mouse	Swiss white	i.p.	–	–	67%	–	–	
*T. congolense*, KETRI 2765	Mouse	Swiss white	i.v.	–	–	50%	–	–	
*T. congolense*, KETRI 2765	Mouse	Swiss white	i.m.	–	–	100%	–	–	
*T. congolense*, KETRI 2765	Mouse	Swiss white	s.c.	–	–	100%	–	–	
*T. congolense*, KETRI 2765	Mouse	Swiss white	i.d.	–	–	100%	–	–	
*T. (Dutonella) vivax*, IL 1392	Mouse	CD-1	i.p.	–	–	–	–	100%	(45)
*T. (Dutonella) vivax*, IL 1392	Mouse	CD-1	s.c.	–	–	–	–	100%	
*T. congolense* (GVR 12/1),	Mouse	CD-1	i.v.	100%	100%	100%	100%	–	(46)
*T. congolense, Trans Mara*, TC13	Mouse	BALB/c	i.p.	–	–	–	100%	100%	(47)
*T. congolense, Trans Mara*, TC13	Mouse	BALB/c	i.d.	–	–	–	0%	0%	
*T. congolense*, IL 3274	Mouse	Swiss white	i.v.	100%	100%	100%	100%	100%	(48)
*T. b. rhodesiense*, EATRO 1886	Cattle	Boran	i.v.	–	100%	–	–	–	(49)
*T. vivax, ETBD-1/ETBS 1*	Cattle	Zebu	i.v.	100%	–	–	–	–	(50)
T. vivax, Y58	Cattle	Zebu	i.v.	–	–	100%	–	–	(51)
T. congolense, TREU 112	Cattle	Holstein	i.v.	100%	–	–	–	–	(52)
*T. evansi*, Olmisor isolate	Goat	East African	i.v.	–	100%	–	–	–	(53)
*T. congolense*, Ea-Tc, IL 1180	Goat	West African Dwarf	i.d.	–	–	100%	–	–	(54)
*T. congolense*, ITC 84	Goat	West African Dwarf	i.d.	–	–	100%	–	–	
*T. congolense*, IL 957	Goat	East African	s.c.	–	100%	–	–	–	(55)
*T. congolense*, IL 958	Goat	Galla	s.c.	–	100%	–	–	–	
*T. congolense*, UTRO 170491-B	Goat	Kigezi	i.v.	–	100%	–	–	–	(56)
*T. congolense*, UTRO 170491-B	Goat	Mubende	i.v.	–	100%	–	–	–	
*T. congolense*, UTRO 170491-B	Goat	Small East African	i.v.	–	100%	–	–	–	
*T. congolense*	Goat	Galla	i.v.	–	100%	–	–	–	(57)
*T. congolense*	Goat	East African	i.v.	–	100%	–	–	–	
*T. congolense*	Goat	Saanaen	i.v.	–	100%	–	–	–	
*T. congolense*	Goat	Saanen x Galla	i.v.	–	100%	–	–	–	
*T. congolense*	Sheep	Merino	i.v.	–	100%	–	–	–	
*T. congolense*	Sheep	Blackhead Persian	i.v.	–	100%	–	–	–	
*T. congolense*	Sheep	Red Masai	i.v.	–	100%	–	–	–	
*T. brucei*, CT 70	Sheep	Yankassa rams	s.c.	100%	–	–	–	–	(58)
*T. vivax*, CT 128	Sheep	Yankassa rams	s.c.	100%	–	–	–	–	
*T. congolense*, GT 12	Sheep	Yankassa rams	s.c.	100%	–	–	–	–	
*T. brucei*, Strain 8/18	Sheep	West African Dwarf	i.v.	100%	–	–	–	–	(59)
*T. vivax*, Zarkwai/84/ NITR/11.1	Sheep	Uda	i.v.	–	100%	–	–	–	(60)
*T. congolense*, Ea-Tc, IL 1180	Sheep	Djallonke	i.d.	–	–	100%	–	–	(54)
*T. congolense*, ITC 84	Sheep	Djallonke	i.d.	–	–	100%	–	–	
*T. b. brucei*	Rabbit	New Zealand white	i.p.	100%	–	–	–	–	(61)
*T. b. brucei*	Rabbit	Chinchilla White	i.p.	100%	–	–	–	–	
*T. rhodesiense, EATRO 1886*	Rabbit	New Zealand white	i.v.	–	–	100%	–	–	(62)
*T. b. gambiense*, MBA ITMAP 1811	Monkey	Vervet	i.v.	–	–	–	100%	–	(63)
*T. b. brucei*, GUTat 1	Monkey	Vervet	i.v.	–	–	100%	–	–	(64)
*T. b. rhodesiense*, KETRI 2537	Monkey	Vervet	i.v.	–	–	100%	–	–	(65)

*i.p., intraperitoneal; i.d., intradermal; i.v., intravenous; i.m., intramuscular; s.c., subcutaneous*.

In addition to infection route, inoculum dose has also been shown to influence the outcome of trypanosome infection (44). For example, goats infected i.d. with *T. congolense* showed a delay in the onset of a local skin reaction (chancre formation) as the inoculum dose decreased (66), with a concomitant decrease in the size of the chancre. Consistent with these observations, a similar effect was reported for BALB/c mice infected i.d. with *T. brucei*, showing reduced infection rates at lower doses compared to higher doses (43). In this study, all animals infected with doses ranging from 1 × 10^5^ to 1 × 10^4^ parasites developed detectable parasitemia. Conversely, doses of 1 × 10^2^ parasites led to no patent infections and 1 × 10^3^ parasites resulted in only 50% of the animals developing a patent infection. In contrast, all the mice infected with 1 × 10^2^ parasites i.p. developed detectable parasitemia at the same time point, indicating that the skin poses a significant barrier for systemic dissemination and infection dynamics. These results suggest that there is a minimal infective parasite dose able to survive the initial challenge mounted by the local immune response in the skin.

The dose and route of infection has been shown to affect the dynamics of several protozoan infections, including the related trypanomastid *Leishmania*. These parasites are similarly transmitted to the skin of the mammalian host through the bites of female sand fly vectors (67), although they differ from extracellular African trypanosomes in that the inoculated lifecycle stage invades mammalian cells and replicates intracellularly. Upon feeding, the sand fly regurgitates metacyclic promastigote forms into the skin that are then phagocytosed by host macrophages. Promastigotes then develop into amastigote forms and replicate within the host cell. Subcutaneous (s.c.) injections of *Leishmania major* and *L. tropica* in mice result in a lower systemic parasite burden and increased protective immunity compared to i.p. and i.d. infections (68, 69). An increased Th1-associated resistance to *Leishmania* infection was induced in BALB/c mice following a low dose of parasite inoculum administered either i.d. or s.c. (69, 70), whereas a high inoculum induced a more Th2-skewed immune response that led to higher susceptibility and systemic parasite burden. These observations in related parasites highlight the importance of the initial parasite dose and site of infection for disease outcome. More importantly, these initial interactions might favor further dissemination (e.g., by infecting recruited immune cells) or the formation of quiescent parasite foci relevant for diagnostics and infection recrudescence upon treatment. It is likely that there are similar effects occurring during the initial skin stages of African trypanosome infections, although the factors determining parasite survival and migration remain to be fully explored.

### Immunity to African Trypanosomes in the Skin

**Figure 2:**
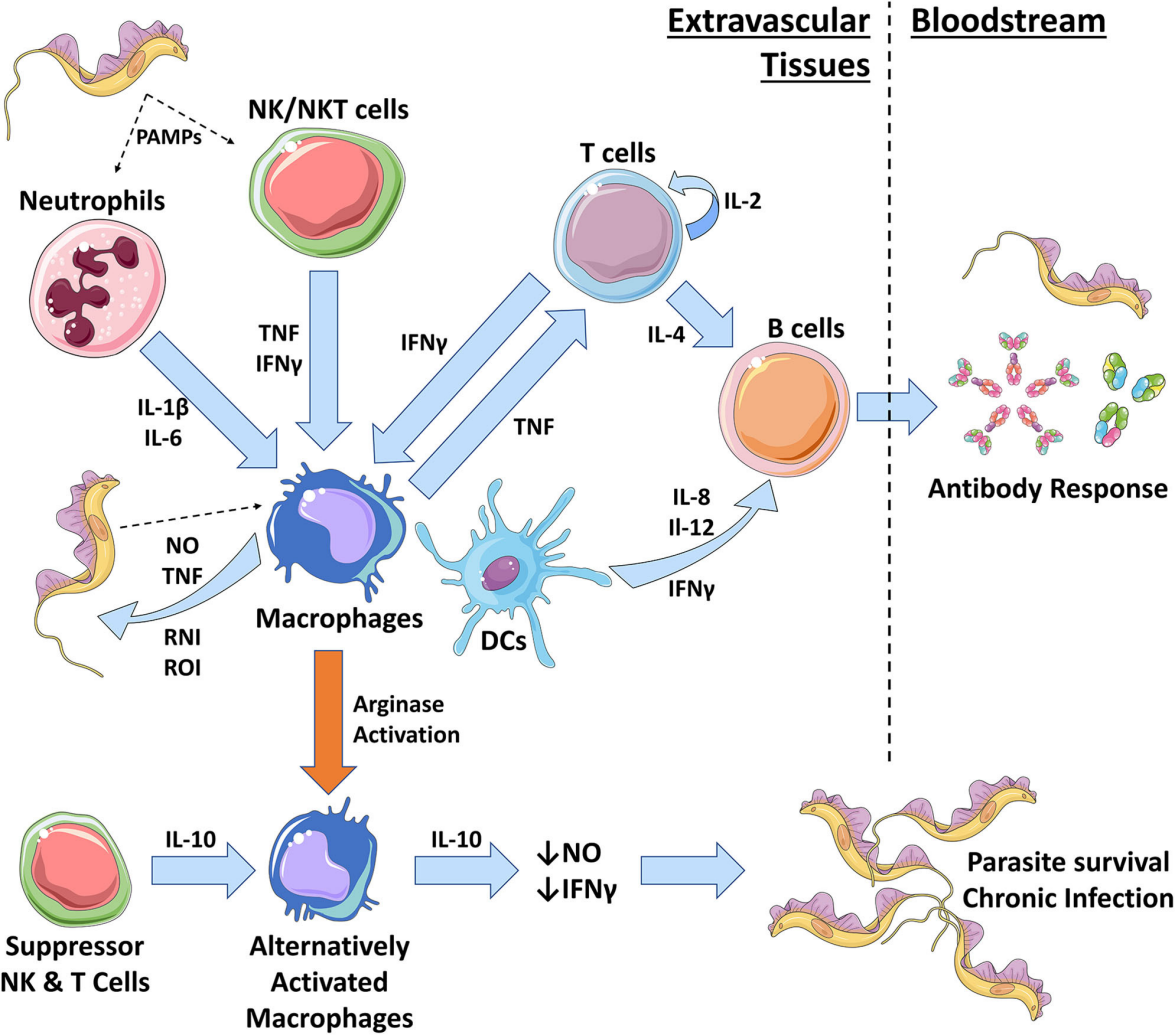
The role of innate immune cells during African trypanosomiasis. During early trypanosome infection, a strong Th_1_ immune response is initiated by the host. In the skin, neutrophils and NK cells are the first to respond to trypanosomal pathogen-associated molecular patterns (PAMPs), such as VSG and CpG DNA. Neutrophils are heavily involved in repairing the initial wound caused by the tsetse fly bite and also produce pro-inflammatory IL-1β and IL-6. NK cells produce pro-inflammatory TNF and IFN-γ that results in the classical activation of pro-inflammatory macrophages (Mθ) via iNOS activation. Macrophages can also be activated through interactions with trypanosomal PAMPs. Classically activated macrophages produce further pro-inflammatory molecules, including TNF, nitric oxide (NO), and reactive nitrogen intermediates (RNIs) and reactive oxygen intermediates (ROIs). These chemicals can directly kill trypanosomes in extravascular spaces and tissues and allows for parasite control. Macrophage secretion of TNF can also recruit and activate T cells which self-renew via autocrine IL-2 secretion. T cells produce IFN-γ to further activate macrophages and IL-4 to activate B cells. Macrophages and dendritic cells (DCs) will further activate B cells during a Th_1_ response, via IL-6, IL-12, and IFN-γ, to promote the production of antibodies that can target the VSG trypanosomes, inducing waves of parasite clearance in the bloodstream. However, antigenic variation hinders the effective clearance of trypanosome populations. Macrophages can also become alternatively activated, resulting in a Th2 immunosuppressive response. Cytokines such as IL-10, IL-4, and TGF-β initiate this type of response by promoting arginase activation in macrophages. As a result, alternatively activated macrophages produce immunosuppressive IL-10 and suppress production of trypanostatic NO and IFN-γ. This promotes parasite growth and survival, leading to a chronic infection.

African trypanosomes exist entirely extracellularly within the mammalian host and are constantly exposed to the host innate and adaptive immune systems. Previous studies examining systemic immune responses in mice using artificial inoculation routes (primarily i.p. and i.v.) have found numerous components that are vital to controlling the initial stages of trypanosome infection, including macrophages, monocytes, dendritic cells, neutrophils, and NK cells (Figure 2) (71–75). The early immune response (<2 weeks) is characterized by an induction of a strong pro-inflammatory profile, including the expression of IFN-γ, tumor necrosis factor (TNF), IL-6, and the production of nitric oxide (NO) (3, 76–82). An adaptive B cell response is also elicited, leading to the production of antigen-specific antibodies that target the immunodominant variant surface glycoprotein (VSG) at the parasite surface. However, the artificial inoculation routes used in these studies overlook the events that occur in the skin early during infection. Through understanding the processes involved in establishing infection in the mammalian host and establishing how the immune system interacts with the parasites in the initial stages, skin-targeted research could provide important information on how the disease progresses within the host. This additional information may reveal methods to develop novel methods for controlling disease transmission in humans and animals. It is also likely that the immune responses in the skin have wider impacts on the systemic infection, similar to those observed in *Leishmania* infections (69, 70) and recent data have shown that there is a population of African trypanosomes present in the skin of the host (11, 18, 83). These parasites are integral to transmission, but their persistence suggests that the parasites can avoid or co-opt the immune response in the skin. Understanding how this is achieved could lead to methods to limit this population, reducing transmission. This skin-dwelling population also presents issues for new therapeutics targeting African trypanosomes that have been developed for parasites in the bloodstream and CNS. Although there has been little work to date on skin immune responses during trypanosome challenge, inferences can be made from systemic studies and related parasites, in addition to the small number of studies that have been performed.

### Neutrophils and Natural Killer (NK) Cells—the First Responders

It is likely that some of the earliest cellular responders to trypanosome inoculation in the skin are neutrophils, NK cells, and NKT cells (3, 84, 85). Neutrophils are some of the most ubiquitous leukocytes in the human immune system and are involved in the killing of many pathogens (including protozoa). They act through phagocytosis, the release of reactive oxygen species, and neutrophil extracellular traps (NETs) (86–89). They are also important mediators of tissue repair and wound healing, producing various pro-inflammatory cytokines that include transforming growth factor-β (TGF-β), IL-4, IL-12, and IL-13 (88). They may also potentially release IFN-γ, although it is unclear whether neutrophils are a true source of IFN-γ, especially in humans (90). As such, our understanding of these mechanisms relies heavily on murine data that may not be applicable to human mechanisms of immunity. In these model experiments, neutrophils have been shown to be the primary responders to tsetse fly bites and are recruited to the dermal bite site within 4.5 h (85). At the same time as neutrophil recruitment, there is an induction of pro-inflammatory IL-1β and IL-6, as well as anti-inflammatory IL-10 (85), from unidentified sources. Neutrophils may also produce trypanolytic antimicrobial peptides, such as cathelicidins and defensins (91), although this early neutrophil response does not appear to contribute to trypanosome killing in the skin (85).

Cytotoxic NK and NKT cells are also commonly employed during the earliest periods of pathogen infection. However, the roles of NK cells during trypanosome infection are poorly understood and investigation is limited to systemic studies. NK cell-deficient mice infected with *T. congolense* are unable to control the levels of parasitemia due to lower levels of IFN-γ and TNF, leading to rapid onset of death due and uncontrollable parasitemia (92). This lethal phenotype was rescued when NK cells were transferred into the NK cell-deficient mice prior to infection. Moreover, mice infected i.p. with *T. congolense* show a systemic recruitment of NK cells that are thought to provide early production of IFN-γ and TNF in the blood, spleen, lungs, and liver (92). NK cell activity in trypanotolerant strains of mice (strains that display reduced clinical disease when infected with trypanosomes compared to susceptible counterparts) infected with *T. congolense* has been suggested to be due to an increase in the production of IFN-γ during infection (72). The activation of neutrophils, NK cells, and NK/T cells in the skin following exposure to trypanosomal pathogen-associated molecular patterns (PAMPs) also results in the production of IFN-γ and TNF that can induce the activation of classically activated macrophages (93). In this context, we speculate that recruitment of NK cells in the skin also provide early protective immunity in trypanotolerant hosts, utilizing pro-inflammatory cytokines to promote further immune activation and parasite killing. Studies regarding the spatio-temporal recruitment of immune effectors to the skin during *T. brucei* infection might shed lights into these processes.

Similar to African trypanosomes, *Leishmania* spp. are transmitted into the subdermal layer of the skin by female phlebotomine sand flies (67, 94). In this case, it has been shown that neutrophils in the skin are the initial responders to infection and phagocytose *Leishmania* metacyclic promastigotes (95). Two-photon intravital imaging has shown that 40–60 min post-infection, a rapid early recruitment of neutrophils is induced at the site of infection in the skin following sand fly feeding (96). Here, the neutrophils occupy the epidermis in large numbers where they directly kill promastigotes using NETs (86, 97) and active phagocytosis of parasites (96). However, there is substantial evidence that promastigotes can persist within neutrophils, escaping effective killing, and potentially taking advantage of neutrophils as a means to continue their life cycle within the mammalian host (98, 99). The promastigotes in the infected neutrophils are in turn phagocytosed by macrophages and dendritic cells where they develop into the amastigote form and replicate (94, 95). This neutrophil recruitment response is similar to that observed following an infected tsetse fly bite (85). However, while direct killing by neutrophils has been shown with *Leishmania* parasites, it has not been shown during African trypanosome infections. One hypothesis is that the rapid recruitment of neutrophils during trypanosome infection occurs in response to the tissue damage inflicted by the tsetse fly and is required for wound repair. In this regard, the initial recruitment of neutrophils upon *T. brucei* infection in mice (*via* i.p.) is prolonged after the initial peak of parasitemia. This is thought to contribute to the suppression of NK, NK/T cells, and both T and B lymphocytes in the spleen at later stages of infection (100). This may be through disruption of the splenic microarchitecture due to significant pro-inflammatory responses. How these immunosuppressive mechanisms relate to early infection in the skin, as well as the activation of the skin-resident immune population, remain unknown and should therefore be an important area for further investigation.

### Macrophages—the Big Players

**Figure 3:**
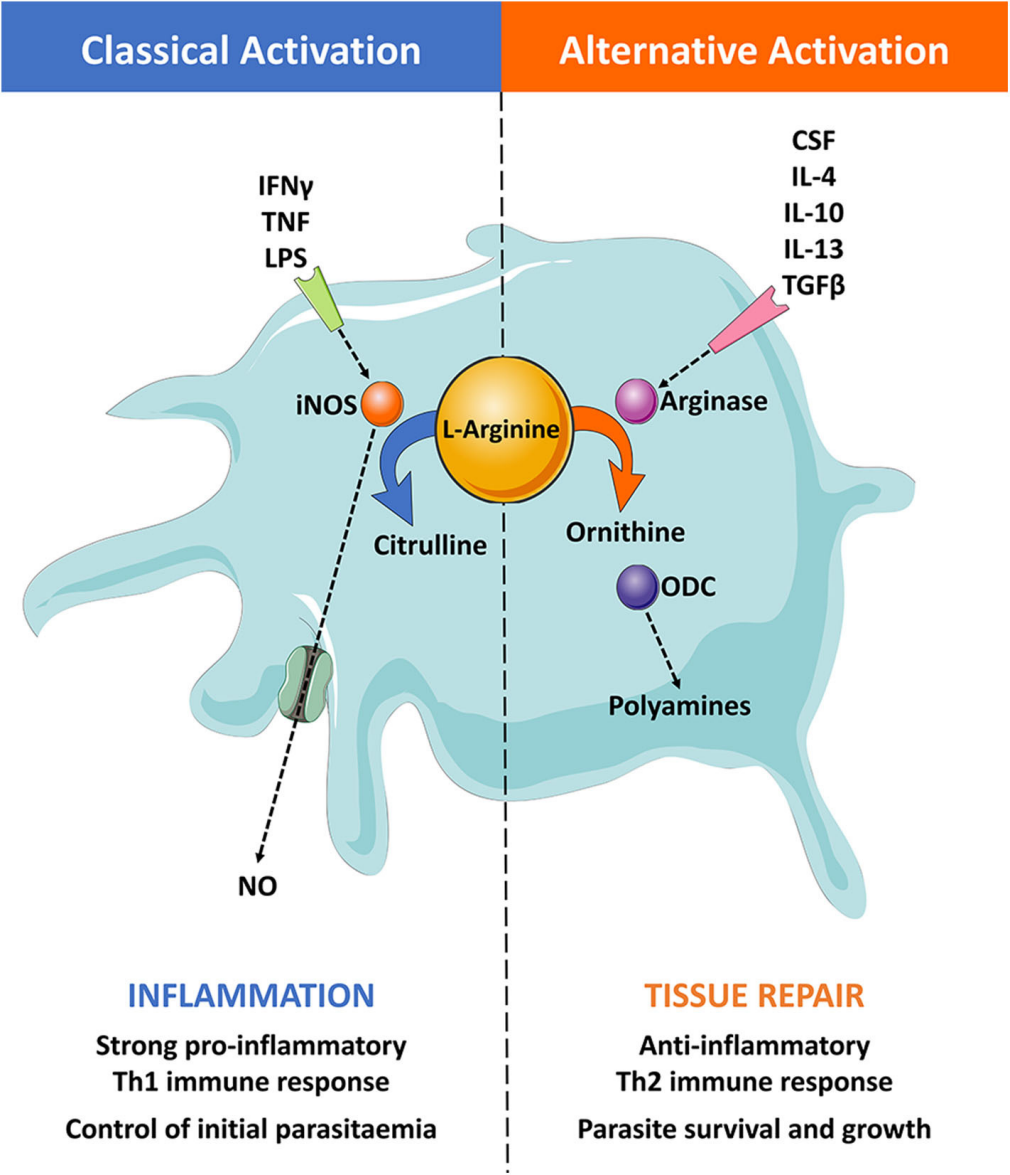
The roles of classically and alternatively activated macrophages. (Left) Pro-inflammatory stimuli, such as IFN-γ, TNF-α, and LPS, classically activates macrophages. Classically activated macrophages induce the expression of the inducible nitric oxide synthase (iNOS) enzyme, which catabolizes the substrate L-arginine to produce NO and citrulline. This results in a pro-inflammatory Th1 immune response that can effectively control the initial parasitaemia peak. (Right) Anti-inflammatory stimuli, such as CSF, IL-4, IL-10, IL-13, and TGF-β, alternatively activate macrophages. These induce the expression of the arginase enzyme that catabolizes the substrate L-arginine to produce ornithine. The enzyme ornithine decarboxylase (ODC) catalyzes the breakdown of ornithine to produce polyamines, resulting in an anti-inflammatory Th2 immune response and tissue repair. This type of response typically leads to an immune environment that promotes trypanosome growth and survival.

The skin contains an abundance of macrophages with the potential to combat infection but again, little is known about the role of skin-resident macrophages during trypanosome infection. During systemic infections, macrophages are considered to play an essential role in combating African trypanosome infections in the mammalian host and are central to mediating the immune response in the extravascular tissues (Figures 2, 3) (3, 74, 101–110). After infection, trypanosome associated PAMPs trigger the activation of these innate mononuclear phagocytes *via* interactions with pattern recognition receptors (PRRs) on the surfaces of host immune cells. These are triggered by parasite-derived molecules such as CpG DNA (recognized by TLR-9) and soluble glycosylphosphatidyl inositol (GPI)-anchored VSG (recognized by scavenger receptor PRRs) (36, 74, 84, 103, 105, 108, 111–115). The release of soluble VSG further stimulates immune cells in a type 1-dependent manner (112). These trigger signaling pathways that lead to the acquisition of a classically activated phenotype in macrophages (and the release of pro-inflammatory cytokines such as IFN-γ and TNF), which is important for quickly controlling invading trypanosomes (Figure 3) (36, 84, 103, 105, 108, 112–114).

The strong pro-inflammatory type 1 (Th1) immune response initiated during the early onset of infection, characterized by elevated levels of IFN-γ and IL-2, leads to an increase in macrophage numbers in the spleen, liver, and bone marrow (106). During clinical disease, macrophage migrating inhibitory factor (MIF) mRNA levels are also increased in the blood of infected patients (116). This could explain the elevated levels of recruited macrophages observed in peripheral immune organs. Macrophages and liver-resident Kupffer cells phagocytose trypanosomes that are opsonized by parasite-specific Ig. These cells are aided by various soluble factors derived from the host complement system (106, 117–119). Experiments using *T. brucei*-infected mice have shown that Kupffer cells in the liver are involved in most of the parasite clearance that occurs *via* complement and antibody-mediated phagocytosis (120). Classically activated macrophages can also utilize the inducible nitric oxide synthase (iNOS) enzyme to produce highly reactive and toxic nitric oxide (NO) *via* the L-arginine metabolic pathway (Figure 3) (121, 122). Additionally, these mononuclear phagocytes can produce further pro-inflammatory cytokines such as TNF, IL-1, IL-6, IL-8, and IL-12 (93). Interestingly, several pro-inflammatory cytokines have been shown to display potent trypanostatic activities, further highlighting the importance of these cytokines during the onset of the infection (3, 72, 84, 123–130). One such trypanostatic effect is believed to be mediated by NO as experimental infections with *T. congolense* and *T. brucei* have shown that NO production can inhibit trypanosome growth and specifically control the first wave of parasitemia (131, 132). However, the role of NO is contentious as *in vivo* studies have reported that NO readily binds to hemoglobin and interacts with red blood cells (76–78). This would mean that NO would be quickly quenched in the bloodstream, removing its inhibitory effect. However, NO may still be effective in the microenvironment of extravascular spaces in which hemoglobin is much reduced or absent, such as the skin (133). Importantly, not all interactions with cytokines appear to be detrimental to trypanosomes. Trypanosomes are known to secrete trypanosome lymphocyte triggering factor (TLTF) that can trigger the production of IFN-γ from CD8+ T cells (134, 135), inducing a potent classically-activated macrophage response. This seemingly contradictory effect may suggest that African trypanosomes can benefit from this specific T cell interaction for survival, or alternatively, that TLTF might play an important role in inducing the development of a more immunotolerant environment for the parasite to sustain survival. This remains speculative but merits further investigation.

The induction of type 2 macrophages is a prominent feature of the immunosuppression that occurs during chronic *T. brucei* infections. Studies have shown that trypanosomes trigger a switch in macrophage activation from pro-inflammatory (or classically-activated) cells to a more anti-inflammatory (or alternatively activated) state (74, 104, 110, 136, 137). This switch in macrophage character skews the host immune response from Th1 to Th2, resulting in an anti-inflammatory environment that promotes parasite survival in the host (138). The implications for this profound switch in immunological state are a matter of active research but the process may contribute to reducing the deleterious effects on the host of sustained inflammation, as well as favoring tissue repair and regeneration (93, 139–143). Macrophages can become alternatively activated through stimulation by macrophage colony-stimulating factor (CSF-1), IL-4, IL-10, IL-13, and TGF-β (Figure 3) (93, 144–146). One of the main hallmarks of alternatively-activated macrophages (AAM) is the expression of arginases upon stimulation, which in turn compete with iNOS and induce ornithine and urea production *via* the L-arginine pathway instead of NO and citrulline (147).

A recent study has also shown that *T. brucei* can actively skew macrophage and glial cell activation by secreting metabolites that suppress their pro-inflammatory functions (148). These macrophage responses varied depending on the strain of the parasite, suggesting it is a parasite-derived factor determining host response, supporting earlier work describing the existence of a parasite-driven virulence factor (149). These trypanosomal factors can trigger macrophages to switch toward a Th2 phenotype and include the *T. brucei* kinesin heavy chain isoform (TbKHC1) that actively induces IL-10 production and arginase activity, resulting in decreased NO production (150). When wild-type mice were infected with TbKHC1 KO trypanosomes, parasitemia was reduced and the survival of the host enhanced (150). These data suggest that the release of TbKHC1 by *T. brucei* enables the parasites to manipulate host cell metabolism by biasing the L-arginine pathway toward arginase enzyme activity, although it remains unclear whether TbKHC1 is constitutively released by viable parasites or is a byproduct from decaying or dead parasites. It has also been suggested that immunosuppression in the skin during intradermal trypanosome infection could be mediated by the combination of a mixed classical/alternative macrophage response and suppressor T cell response (151). These findings suggest that a pro-inflammatory macrophage/Th1 response is needed for the effective control of trypanosomes in the skin, although a Th2 response seems necessary to sustain host survival in the face of a chronic infection. As such, these specific macrophage-trypanosome interactions should be investigated thoroughly for opportunities for blocking disease progression.

### Dendritic Cells—Primers of Adaptive Immunity

Dendritic cells (DCs) are a group of antigen-presenting cells (APCs) that recognize and capture antigen for presentation to T cells (152, 153). Studies using i.p. *T. b. rhodesiense* infections in mice have shown that splenic DCs may be the primary APCs involved in activating VSG-specific T helper (Th) cell responses in trypanotolerant mouse lines through the upregulation of co-stimulatory receptors, the presentation of VSG peptide antigen, and the production of IL-12 (154, 155). However, subsequent VSG antigen processing and presentation was notably reduced during high parasite burdens in the mice, suggesting a trypanosome factor may be interfering with the ability of APCs to process and present antigen to T cells. Alternatively, there may be a potential impairment of the antigen presentation capacity or DC maturation during chronic infection. This remains speculative for African trypanosome infections but has been described for other infections associated with immunotolerant states (156, 157).

Skin-residing DCs include epidermal Langerhans cells and dermal DCs (158–160). Langerhans cells sample and present antigen from the epidermis to promote the adaptive immune response (161, 162). They have been shown to have a suppressive function in *Leishmania* infections by modifying the behavior of regulatory T cells (Tregs) but their role in Africa trypanosomiasis is completely unknown and remains to be established (163). Langerhans cells and dermal DCs migrate from the epidermis and dermis, respectively, to the local cutaneous draining lymph nodes to present sampled pathogen antigen to T cells (158–160, 162). Dermal DCs have also been shown to act rapidly to dermis invading protozoan parasites such as *L. major* (164). It has previously been reported in *L. major*-infected mice that epidermal Langerhans cells localized at the sand-fly bite site become activated and upregulate major histocompatibility complex molecules and co-stimulatory receptors (165). This results in cytokine release (IL-12) and the promotion of a Th1 cellular response. African trypanosomes have recently been shown to form active foci in the skin and it is likely that this would be modulated and controlled by resident DCs and Langerhans cells, similar to related trypanomastids (11, 83). Moreover, DCs might elicit a more immunotolerant state in the skin, ultimately allowing the persistence of skin foci. However, the specific roles of skin resident DCs during trypanosome infection remain to be elucidated.

### T Cells—Surveying the Damage

Skin-resident T cells are another group of immune cells that survey the tissue for pathogens. The epidermal layer is patrolled by αβ effector T cells and more innate-like γδ T cells, in addition to group 2 innate lymphoid cells (ILC2s) (166–168). The γδ T cell population act with innate cells to survey tissue during the early stages of infection before the more conventional adaptive immune cells become involved. Dermal γδ T cells also express many receptors and cytokines that can alter Th1 and Th17 responses and affect extracellular pathogen, including IFN-γ, TNF, and IL-17 (169–171). ILC2s are dependent on IL-7 and constitutively secrete IL-13, thought to be central for interaction with granulocytes (167). Although T cells during skin diseases have been well-studied, particularly for psoriasis and allergies (172–174), the roles of specific γδ T cells, ILC2s, and their associated cytokines during human trypanosome infections are unclear. A study using cattle found that tsetse-transmitted *T. congolense* infection leads to the increased numbers of γδ T cells and that these cells are activated in the trypanotolerant N'Dama breed but not the more susceptible Boran breed (175). However, cattle and other ruminants possess a substantially higher proportion of γδ T cells in the peripheral blood mononuclear lymphocyte population (15–60%) than both humans and mice (<5%) (176) and their role may differ between these different hosts. Importantly, due to the induction of AAM activity observed during chronic infection, a reduction in IL-2 secretion and expression of the α-chain of the IL-2 cytokine receptor in lymph node T cells leads to inhibition of immune responses during *T. brucei* and *T. congolense* infections in mice and cattle (177–181).

While it is unclear what the potential role of skin-resident T cells may be during African trypanosome infection, insights could be gained from the pathogenesis of other parasites. For example, during infection with *Plasmodium* spp., sporozoites induce rapid immunosuppression in the skin (182–184) that affects both T and B cell functionality. *Plasmodium*-specific Tregs are also induced in the skin that expand upon re-exposure to *Plasmodium* antigens and suppress immunity to infection (185). This was found to be linked to parasite specific factors and when the sporozoite surface protein CSP was injected into the skin at low doses with CpG DNA, *Plasmodium*-specific CD8^+^ effector T cells were significantly inhibited (182). This is hypothesized to involve regulatory B cells (Bregs) that produce immunosuppressive IL-10 and TGF-β as depletion of B cells rescued effector T cell function during malaria infection (186). Similarly, it is possible that the suppressive phenotypes observed during African trypanosomiasis also occur in the skin, hindering the successful elimination of trypanosomes. Indeed, very little inflammation or other immune responses were observed in the skin of mice with heavy burdens of skin-dwelling *T. brucei* parasites (83).

### Lymphatic Invasion and Systemic Dissemination

Following skin infection, trypanosomes trigger a series of immunological events that activate resident immune cells and promote recruitment to the site of infection. This in turn delineates long-term disease outcome, specifically by determining how the parasite will disseminate systemically and establish infection. In order to achieve this, trypanosomes must overcome the initial immunological challenge mounted in the skin and travel to the afferent lymphatics, entering the local draining lymph nodes (Figure 1) (11, 14–17, 151). Historical studies using dogs found that *T. brucei equiperdum* leaves the dermis via the afferent lymphatics, spreading into the draining lymph nodes before reaching the bloodstream (15). A similar sequence of events has also been shown in cattle, goats (14, 187), and recently mice (11). In this recent mouse study, trypanosomes were first detected within the local draining lymph nodes prior to their detection in the bloodstream (18 h compared to 42 h post-infection, respectively) (11). Intravital imaging of infected mice following tsetse fly bites of the skin has also been used to elucidate some of these mechanisms (17). In these experiments, a large number of parasites exhibited directional migration following trypanosome injection into the skin, traveling back and forth toward the lymphatic vessels. In addition to parasites found in the skin interstitium, parasites were also found within the afferent lymphatic vessels of the dermis. These parasites were characterized by significantly higher velocities than their extravascular counterparts, suggesting behavioral diversity. This is similar to the diverse ranges in parasite motility previously described for *T. carassii* in zebrafish (188). This would indicate that there are unknown mechanisms through which African trypanosomes are attracted toward and then invade the afferent lymphatics of the skin, allowing their subsequent systemic dissemination in the host (Figures 1B,C). Similar to African trypanosomes, large numbers of *Plasmodium* sporozoites have been shown to remain in the dermis while others drain to the local lymph nodes (189). These skin-resident sporozoites “glide” through the dermis and can invade the local dermal blood and lymphatic vessels before reaching the liver (190–192). Imaging studies in rodents have shown that skin sporozoites largely drain *via* the lymphatics having been phagocytosed by dendritic cells. Conversely, a minority enter directly into the bloodstream (193). Intravital imaging has shown that immunization of mice with attenuated sporozoites and *P. berghei* circumsporozoite protein inhibits sporozoite motility in the skin, resulting in inhibition of dermal blood vessel invasion (194). In addition, *Leishmania* parasites are known to form reservoirs in the skin that enhance their onward transmission to the sand fly vector (195). However, several species of *Leishmania* also disseminate systemically around the host and this has been suggested to involve infected macrophages or dendritic cells carrying the parasites to the local draining lymph nodes (94). Although African trypanosomes do not invade cells in the host, it is possible that they may be chaperoned into the local draining lymph nodes *via* macrophages or dendritic cells, either through an unknown attachment mechanism or a chemical cue.

It is also plausible that lymphatic accumulation may be driven by hydrostatic pressure, protein and/or chemical gradients, or the sensing of lymph flow (196–201). These environmental cues could direct trypanosomes toward open junctions in the lymphatic epithelium, similar to the systems used by lymphocytes for lymphatic invasion (199, 202, 203). For example, dendritic cells have been shown to respond to gradients of CCL19 and CCL21 chemokines expressed in the lymphatic vessels (204), facilitating entry into the lymphatics in the skin (205), and CXCL12 gradients have been shown to be important for the initiation of dendritic cell responses in the skin (206). However, there was no evidence for the role of several host-derived chemokines in attracting trypanosomes in a recent study (17). As African trypanosomes possess chemosensory capabilities through receptors found on the flagellum and flagellar pocket (207), it is feasible that they could respond to non-chemokine chemical gradients within the host to reach the lymphatics. For example, glucose is crucial for the metabolism of bloodstream form trypanosomes (208) and glucose concentrations in canines have been shown to be higher in the lymph than the blood (209). Glucose, lipids, or other factors essential for trypanosome metabolism, could therefore act as chemical chemoattractants for trypanosomes. The identification of potential these (tissue-specific) parasite chemoattractants merits further investigation as these might be key for understanding tissue colonization and the development of transmission-inhibiting therapeutics. Recent *in vivo* imaging has also shown that the presence of a hydrodynamic flow impacts substrate binding and swimming in trypanosomes, suggesting that the forces acting on these parasites can directly lead to changes in behavior that promote dissemination (188). Regardless of mechanisms involved, two central questions remain: (i) what are the processes deployed by trypanosomes to circumvent the immunological and physical barriers that they encounter in the skin *en route* to the lymphatic system? And (ii) are these interactions established by direct cell-cell contact between trypanosomes and host cells, or mediated by secreted factors (e.g., soluble virulence factors or extracellular vesicles)? An intriguing hypothesis is that upon differentiation, the proliferative long-slender trypomastigotes, in addition to immune cells recruited to the site of infection (e.g., neutrophils and macrophages), may actively remodel tissue architecture in a manner that facilitates movement from the site of infection and the establishment of a systemic infection. Understanding these mechanisms could again lead to tools that disrupt these behaviors, affecting transmission and the establishment of systemic infections in humans and animals.

In summary, lymphatic tropism leads to the accumulation of trypanosomes within the lymph nodes, triggering a myriad of adaptive immune responses before systemic dissemination (17, 94, 189–195). Ultimately, entry into the lymphatic system enables direct access to the bloodstream as fluids and cells drain through the thoracic duct and right lymphatic ducts re-joining the systemic circulation via the subclavian veins. Dissemination of the parasite and continued interaction with the host immune system would drive many of the consequent pathologies associated with infection. Recirculation through the dermal capillary beds also presents accessible trypanosomes to infect the tsetse fly vector, facilitating transmission and disease persistence. However, recent evidence has emerged of extravascular, skin-dwelling parasites that are also involved in transmission (11, 18, 83). Understanding this new anatomical niche has therefore become key to ongoing efforts to control the disease, particularly in the context of recently described latent HAT infections that may also be infective (210, 211).

### Trypanosome Reservoir in the Skin

Historically, the presence of African trypanosomes in the host skin was a widely recognized aspect of infection that has been largely supplanted by the repeated description of *T. brucei* as a bloodstream parasite (212). Re-discovering this overlooked anatomical reservoir, and the potential implications for transmission, treatment, and control, has become a focus of trypanosome research (213). This reassessment includes the description of a metabolically unique population of *T. brucei* parasites found in the adipose tissues of various organs in the mammalian host (18). These adipose tissue form (ATF) parasites metabolize fatty acids through β-oxidation and utilize the tricarboxylic acid cycle rather than glycolysis, making them more similar to the procyclic forms found in the tsetse fly midgut rather than mammalian bloodstream forms (214). While this study did not directly find ATF trypanosomes resident in the skin, the large deposits of subcutaneous adipose (particularly in non-murine hosts) would make it an ideal environment for the parasites. Further studies have since confirmed that *T. brucei* parasites are indeed present in the skin, both interacting with adipocytes (11) and throughout the extravascular space between the panniculus carnosus and the dermis (83). However, it is currently unclear whether these skin-dwelling parasites are ATF or transcriptionally distinct.

Nevertheless, video evidence demonstrates that skin-resident African trypanosomes are motile and undergo division (83, 215). There is little overt inflammation associated with trypanosome numbers in the skin (83), although there is a rise in temperature that may serve to attract tsetse flies to sites of infection (11). Within the skin, the number of parasites cycles with the characteristic peaks and troughs associated with trypanosome infections but the cycle does not appear linked to numbers in the blood, suggesting limited transfer between compartments (83). The extent of parasite exchange between the extravascular compartment and the blood is an aspect of infection that requires further study as this has implications for treatment and pathogenesis. Skin-dwelling parasites also proceed through their life cycle and develop into characteristic “stumpy” forms that are pre-adapted to survive in the tsetse fly vector (216). The presence of stumpy trypanosomes was unequivocally demonstrated via the detection of the stumpy-specific marker PAD-1 (217) in parasites in the skin (83). Importantly, these extravascular trypanosomes contribute to tsetse fly transmission, revealing that the skin is not a “dead-end” for this supposed blood parasite. Initially, a study involving dual infection with two fluorescently tagged trypanosome strains used RT-qPCR quantification to establish that tsetse flies were predominantly infected by parasites resident in the skin rather than the blood (11). Subsequent experiments comparing infectivity with tsetse flies fed on patches of mouse skin, either with or without tissue resident trypanosomes, showed that blood and skin parasites contribute to infection, with both required for maximal transmission (83). These experiments also showed that tsetse flies could be infected by skin-resident trypanosomes in apparently aparasitemic hosts.

As HAT approaches elimination in humans, there has been an increased emphasis on understanding how the disease avoided elimination in the past. In addition to animal reservoirs (218, 219), the role of asymptomatic or latent human infections has begun to receive attention (210, 211). Counter to decades of dogma, field studies have now shown that there are individuals able to tolerate *T. b. gambiense* as latent infections without developing symptoms (211). This latent period can be extremely protracted, with a patient recently described who was infected for at least 29 years without symptoms (220). Latent individuals rarely display detectable blood parasites and are diagnosed via serology (211). However, murine experiments demonstrating that skin-dwelling trypanosomes can infect tsetse flies raise the possibility that these latent infections can contribute to transmission and act as a reservoir of infection. Recent predictive modeling also indicates that aparasitemic but infective hosts are required for a disease focus to be stable in the absence of an animal reservoir (221). This hypothesis is supported by xenodiagnosis experiments showing tsetse flies can be infected by apparently aparasitemic hosts (222, 223) and the identification of trypanosomes in historical skin samples from HAT foci (83). To date, there has been no thorough examination of trypanosomes in the skin of domestic or peri-domestic animals and this large potential reservoir requires further study to fully understand the impact on both human and animal disease.

In summary, the notion that African trypanosomiasis is a disease of just the lymphatic and circulatory systems is being re-assessed. There is strong evidence for skin-resident parasites prior to and long after systemic parasite burden, with the potential for transmission from apparently asymptomatic hosts. This has wider impacts for understanding pathogenesis, developing new therapeutics, and identifying overlooked reservoirs. These field and laboratory data also suggest that overlooked parasites in the skin of human and animal reservoirs threaten the WHO goal of eliminating HAT transmission by 2030 (218). Fully understanding the role of the skin as a biological niche for African trypanosomes will therefore likely continue to shape the field of African trypanosomiasis research.

## Concluding Remarks

African trypanosomes have evolved sophisticated mechanisms to swiftly adapt to rapid changes in their microenvironment, like those encountered by metacyclic trypomastigotes delivered by the tsetse fly in the vertebrate skin when taking a blood meal. These changes are not only physical (e.g., changes in temperature and oxygen pressure) but also mechanical (e.g., transitioning from tsetse fly saliva to solid tissues such as the skin), and immunological (e.g., activation and/or recruitment of immune cells upon infection). In this scenario, it seems plausible that a combination of extrinsic factors, such as those encountered in the skin, exerts a selection pressure for trypomastigotes that are able to overcome these barriers when migrating to nearby lymphatics, leading to the establishment of systemic infections. The skin is therefore the natural point of first contact between trypanosomes and hosts, playing an active role in infection establishment and disease outcome. However, several questions remain unanswered. For example, it is unclear whether metacyclic trypomastigotes use different environmental cues in the skin as drivers for differentiation, and if so, what the chemical nature of these differentiation signals may be. It also remains to be determined whether the initial parasite population at the site of infection remains in the skin, forming a skin-resident parasite subpopulation that is distinct from the parasites found in other tissues and organs (e.g., bloodstream forms), or whether the skin is colonized multiple times as a results of parasite migration to and from the bloodstream.

From the perspective of host-pathogen interactions, it is clear that trypanosomes release a myriad of virulence factors, including soluble products and extracellular vesicles, thought to be critical to modulate the immune response against them (224–228). In this case, it would be important to understand whether the secretion of virulence factors differs between metacyclic and long slender trypomastigotes and to what extent these secreted molecules aid in the establishment of chronic infections in the skin. Similarly, it is still unclear whether the local immune response in the skin at the site of infection helps shape the population structure of parasites that systemically disseminate within the host, and the mechanisms involved in the activation of resident skin immune cells (e.g., Langerhans cells and γδ T cells). The application of novel approaches to identify cellular heterogeneity (e.g., single cell transcriptomics and spatial transcriptomics) would help to clarify the series of events that take place in the skin before parasite dissemination; from parasite differentiation and diversity, to the activation of resident and recruited immune cells.

Finally, it is now clear that the parasites residing in the skin are central to disease transmission and present in both murine models and human clinical samples. Screening of the skin in both humans and animals is likely required to fully understand the true extent of African trypanosome infections in the field, especially as latent infections are likely still infective to tsetse flies due to skin-dwelling parasites. However, our understanding of the skin as an active immune organ delineating disease outcome in human African trypanosomiasis is in its infancy, and we anticipate that future studies should address a myriad of key basic questions in this novel field of research. As such, we need to better understand the specific mechanisms involved in establishing infection in the skin, parasitic migration from the skin, and their subsequent invasion of the lymphatics that leads to systemic infection of the host. It is also important that newly developed drugs targeting the parasite can enter the skin and remain functional, otherwise transmission will be maintained alongside increasing treatment failures as skin-dwelling parasites are selected for. In addition, it is also important to understand the host-parasite interactions that are occurring in the skin and whether they can be manipulated to act as a potential therapeutic or transmission limiting tool? How are trypanosomes reaching the lymphatics from the skin and can they be inhibited from doing so? These are all questions that need to be addressed to if we are to better understand the pathogenesis of African trypanosomiasis and develop new methods of controlling and limiting disease transmission in humans and animals.

## Author's Note

The figures use art adapted from material provided by Servier Medical Art under a Creative Commons Attribution 3.0 Unported License (https://creativecommons.org/licenses/by/3.0/).

## Author Contributions

OA, JQ, AM, PG, RB, JB, NM, LM, and PC drafted and edited the manuscript. All authors approved the final version of the manuscript.

## Conflict of Interest

The authors declare that the research was conducted in the absence of any commercial or financial relationships that could be construed as a potential conflict of interest.
