# Corrigendum: To the Skin and Beyond: The Immune Response to African Trypanosomes as They Enter and Exit the Vertebrate Host

**DOI:** 10.3389/fimmu.2021.780758

**Published:** 2021-10-27

**Authors:** Omar A. Alfituri, Juan F. Quintana, Annette MacLeod, Paul Garside, Robert A. Benson, James M. Brewer, Neil A. Mabbott, Liam J. Morrison, Paul Capewell

**Affiliations:** ^1^ Roslin Institute, Royal (Dick) School of Veterinary Studies, University of Edinburgh, Edinburgh, United Kingdom; ^2^ Wellcome Centre for Integrative Parasitology, Institute of Biodiversity, Animal Health and Comparative Medicine, College of Medical, Veterinary and Life Sciences, University of Glasgow, Glasgow, United Kingdom; ^3^ College of Medical, Veterinary and Life Sciences, Institute of Biodiversity, Animal Health and Comparative Medicine, University of Glasgow, Glasgow, United Kingdom

**Keywords:** African trypanosomiasis, *Trypanosoma brucei*, skin, transmission, innate immunity, neglected tropical disease

In the original article, there was a mistake in **Figure 1** as published. Panel C had an incorrectly orientated lymph node schema. The corrected **Figure 1** appears below.

**Figure 1:**
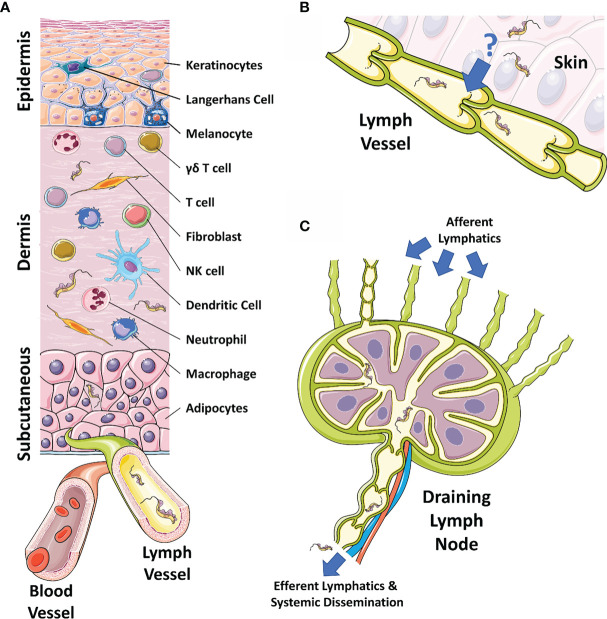
The skin, draining lymphatics, and lymph nodes. **(A)** Diagram of the cellular composition of the epidermal, dermal, and subcutaneous layers of mammalian skin. The outermost epidermal layer consists of a layer of corneocytes above a layer of keratinocytes. These cells manage the tight junctions and the stratum corneum. Langerhans cells and intraepithelial T cells survey the epidermis for antigen to be presented. The central dermal layer contains fibroblasts that produce extracellular matrix proteins to provide structural support and elasticity. Immune responses are initiated by dermal macrophages, dermal dendritic cells, NK cells, and T cells. The inner subcutaneous layer primarily consists of adipocytes. Local lymphatic and blood vessels allow for the trafficking of cells, proteins, and waste. The initial tsetse fly bite injects trypanosomes into the dermis. From the dermis, the parasites exhibit tropism that leads to migration toward the afferent lymph vessels in the skin disseminating to the blood and other regions of the body. **(B)** The mechanism behind directional migration of trypanosomes from the skin to the lymphatics is unknown. Parasites may be responding to an unreported chemical cue in a chemotactic manner and they may crawl along lymph vessels, access open junctions, or are drawn into the lymphatics through hydrodynamic flow force and pressure. **(C)** Afferent lymphatic vessels in the skin allow for the drainage of leukocytes and antigen into the draining lymph node. Lymph, containing activated T and B cells, plasma cells, and antibody, passes into the medullary sinus, before exiting via efferent lymphatic vessels. Trypanosomes enter the draining lymph nodes, causing lymphadenopathy, and exit via the efferent lymphatics. Systemic dissemination of the host is reached via the main lymphatic ducts. The figure uses art adapted from material provided by Servier Medical Art under a Creative Commons Attribution 3.0 Unported License (https://creativecommons.org/licenses/by/3.0/).

The authors apologize for this error and state that this does not change the scientific conclusions of the article in any way. The original article has been updated.

## Publisher’s Note

All claims expressed in this article are solely those of the authors and do not necessarily represent those of their affiliated organizations, or those of the publisher, the editors and the reviewers. Any product that may be evaluated in this article, or claim that may be made by its manufacturer, is not guaranteed or endorsed by the publisher.

